# Utility of early, short psychological care for women who experience early miscarriage: protocol for the randomized, controlled MisTher trial

**DOI:** 10.1186/s40359-023-01421-x

**Published:** 2023-11-03

**Authors:** Coralie Barbe, Marie Boiteux-Chabrier, Emilie Charillon, Leïla Bouazzi, Chloe Maheas, Fadela Merabet, Olivier Graesslin, Julie Auer, Sabrina Hammami, Anne-Catherine Rolland, Aline Hurtaud

**Affiliations:** 1 Comité Universitaire de Ressources pour la Recherche en Santé, Université de Reims Champagne-Ardenne, UFR Médecine, 51 rue Cognacq Jay, 51100 Reims, France; 2 Laboratoire C2S (Cognition, Santé, Société), EA6291, Université de Reims Champagne Ardenne, 51100 Reims, EA France; 3 Département de Médecine Générale, Université de Reims Champagne-Ardenne, UFR Médecine, 51 rue Cognacq Jay, 51100 Reims, France; 4 UFR Médecine, 51 rue Cognacq Jay, 51100 Reims, France; 5 Service de Gynécologie et d’Obstétrique, Centre Hospitalier Universitaire de Reims, Avenue du Général Koenig, 51100 Reims, France; 6 Service de Psychothérapie de l’enfant et de l’adolescent, Pôle Femme-Parents-Enfant, Centre Hospitalier Universitaire de Reims, Avenue du Général Koenig, 51100 Reims, France

**Keywords:** Early miscarriage, Psychological care, Anxiety, Depression, Post-traumatic stress disorder

## Abstract

**Background:**

Around one in ten women will have a miscarriage in their lifetime. Miscarriage is often considered a trivial event by caregivers, but it is associated with a high burden of psychological morbidity, especially during the first 6 months. There is no validated psychological management strategy for women who have had a miscarriage. The MisTher study aims to evaluate the utility of early, short psychological care for women who have had early miscarriage, in terms of anxiety, depression and post-traumatic stress disorder.

**Methods:**

This is a prospective, multicenter, randomized, controlled, superiority study. In total, 932 women who have experienced early miscarriage (spontaneous interruption of pregnancy prior to 14 weeks of gestation) will be randomly assigned to either the intervention or the control group. The intervention consists of 4 teleconsultations of 45 min with a psychologist. All women, regardless of their allocated group, will be encouraged to seek an early consultation with a general practitioner or midwife. The primary endpoint will be anxiety at 3 months after randomization evaluated using State Trait Anxiety Inventory. The secondary endpoints will be anxiety at 6 months evaluated using State Trait Anxiety Inventory, depression at 3 and 6 months evaluated with the Beck Depression Inventory, and post-traumatic stress disorder at 3 and 6 months, evaluated using the Posttraumatic stress disorder Checklist Scale.

**Discussion:**

This project will validate the importance of early psychological management, based on primary care and accessible to most women, via teleconsultation, in reducing the frequency of psychological disorders after early miscarriage. Our results should provide a basis for new recommendations for the management of women who have experienced miscarriage, notably by recommending the involvement of trained psychologists in the management pathway for these women.

**Trial registration:**

The trial is registered with ClinicalTrials.gov: NCT05653414. December 15th, 2022.

## Background

Miscarriage is defined as the spontaneous loss of pregnancy before the foetus has achieved viability. The French national college of gynaecologists and obstetricians (Collège national des gynécologues et obstétriciens) defines early miscarriage as the spontaneous expulsion of an inter-uterine pregnancy at less than 14 weeks gestation [1]. The occurrence of early miscarriage complicates more than 10% of pregnancies [1]. According to Quenby et al., early miscarriage accounts for 15.3% (95% confidence interval (CI) 12.5–18.7%) of diagnosed pregnancies [2]. The prevalence of women who have experienced a miscarriage is 10.8% (95%CI 10.3–11.4%), and overall, it is estimated that 23 million miscarriages occur annually worldwide [3].

Because of its frequency, miscarriage is often considered a trivial event. However, for women who experience miscarriage, it can be a traumatic event associated with grief [4]. In a systematic review by Mergl et al. that included 13 cross-sectional and 8 longitudinal studies totalling 2,597 patients from 11 countries, 17 of the 21 studies observed marked grief in women after a miscarriage or stillbirth [5].

In a paper published in 2016, Markin discusses some common errors made in the treatment of perinatal grief after a miscarriage, including minimizing or avoiding painful affects related to the miscarriage, assuming that the patient’s grief will be resolved in case of a subsequent healthy pregnancy, and neglecting early unresolved losses that are reawakened by the miscarriage [6]. The international literature is in agreement that there is significant psychological morbidity associated with miscarriage. Anxiety, depression and post-traumatic stress disorder PTSD have all been studied in women after miscarriage. According to a review of the literature by Farren et al. in 2018, anxiety is the most common and most persistent psychological disorder after miscarriage [7]. Immediately after a miscarriage, 41% of women present diagnostic criteria for anxiety, with significantly higher scores than in a peripartum control group [8, 9], while at one month after miscarriage, the proportion of women with anxiety was also found to be higher than in a control group [10]. In reported studies, anxiety scores tend to normalize after around 6 months, compared to non-pregnant women [11–13]. However, anxiety may persist up to 12 months after miscarriage, compared to women who had a live birth [7, 14]. Depressive syndrome is diagnosed within 2 weeks after miscarriage in 22 to 36% of women across various studies [8, 12, 15, 16]. Similarly, in women who have had a miscarriage, the relative risk of having a diagnosis of depression was 3 to 4 times higher than in a control group of non-pregnant women [7, 15–17]. The risk of depression is thought to decrease progressively over the first month, although Bergner et al. found that depressive symptoms increased during the first trimester after miscarriage [18]. Finally, PTSD was identified in 25% of women at one month after miscarriage, and in 6% at 4 months in another study [19]. In a study by Farren et al. reported in 2020, at 9 months after a miscarriage, the proportion of women with psychological disorders was 17% for anxiety, 5% for depression, and 16% for PTSD [20].

The psychological troubles associated with miscarriage can have a negative impact on subsequent pregnancies. Fertl et al. reported that women who had a history of spontaneous pregnancy loss had higher levels of anxiety during the first trimester of subsequent pregnancy, compared to women with no history of miscarriage [21]. In a French study by Barbe et al., it was found that during the first trimester of pregnancy, women with a history of spontaneous miscarriage had higher stress levels relating to the medical and obstetric risks or fetal health, than women with no history of miscarriage [22]. Similarly, Haghparast et al. showed that pregnant women who had a history of spontaneous abortion during the previous year had higher scores on anxiety and depression scales (amongst others) compared to pregnant women without a history of spontaneous abortion [23].

The literature surrounding the psychological repercussions of miscarriage does not provide a consensus as to the optimal management of these disorders. A series of articles published in *The Lancet* in 2021 called for a review of data on spontaneous miscarriage, while the accompanying editorial deemed that research into the prevention and management of spontaneous miscarriage should be given priority, including the repercussions for the mental health of the women affected [3]. Similarly, a review of the literature by Coomarasamy et al. concluded that there is a compelling need to screen for psychological difficulties in women who have experienced pregnancy loss [24]. A summary of recommendations also called for the development of strategies to manage the risks associated with miscarriage, notably the mental health consequences [2]. A Cochrane systematic review published by Murphy et al. in 2012 included only 6 randomized, controlled trials that studied non-pharmacological interventions to improve the mental well-being of women who had experienced a miscarriage [25]. The authors concluded that there was insufficient evidence to demonstrate that psychological support was effective after miscarriage, due to the methodological limitations of the studies included (heterogeneity of the psychological follow-up proposed, variation in endpoints, and small sample sizes). In these studies, the psychological interventions studied were heterogeneous, comprising for example a 50-to-60 min psychological consultation or debriefing session [26–28], two to three sessions of one hour of counselling [17, 29], or interventions combining support with self-care advice [26]. Interventions were proposed between 0 and 11 weeks after miscarriage [29, 30], and were delivered by psychologists [27, 28], midwives [26] or specialized nurses [29, 30], either during a consultation or at home. Study endpoints were measured at varying timepoints, ranging from one month [17] to 12 months after miscarriage [29]. The measurement scales used also varied across studies, and included the 12-item General Health Questionnaire (GHQ-12), the Beck Depression Inventory (BDI), the Dyadic Adjustment Scale (DAS) [17], the Hospital Anxiety and Depression Scale (HADS) [27, 28], or the Perinatal Grief Scale (PGS) [31], amongst others. In a systematic review of the literature investigating the effect of interventions to reduce stress, anxiety or depression in pregnant women with a history of miscarriage, San Lazaro Campillo et al. could not find a single study up to 2016 that met their inclusion criteria [32]. Yet, cohort studies and clinical trials suggest that psychological interventions and specific support interventions could improve well-being in women who experience miscarriage, and could mitigate the mental health repercussions on subsequent pregnancies. Barat et al. showed that a 2-hour brief supportive psychotherapy performed during the first 24 h of hospitalization was effective in significantly reducing the frequency of anxiety symptoms (13.5% vs. 60.5%), depressive symptoms (32.4% vs. 71.1%), and grief symptoms (10.8% vs. 65.8%) in the group receiving psychotherapy compared to the control group at four-months of follow-up [31]. A study by Johnson et al. in 2015 suggested that a secondary bereavement intervention on grieving in women who experienced miscarriage at 12–20 weeks gestation promoted women’s ability to cope with early pregnancy loss [33]. A randomized study among 106 Iranian women showed a significant reduction in measures of anxiety (7.9 ± 1.07 vs. 13.79 ± 5.36), stress (9.26 ± 1.25 vs. 18.13 ± 7.66) and depression (7.83 ± 1.05 vs.16.26 ± 11.06) after 8 counselling sessions with mindfulness-based stress reduction techniques, as compared to a control group [34]. At the level of healthcare systems, the World Health Organization (WHO) recommends socioculturally relevant, respectful and dignified care, as well as continuity of care through community-based care providers, such as midwives, to help women deal with pregnancy loss [35].

Currently, in France, there are no specific recommendations for the medical and psychological management of miscarriage in primary care. The regular follow-up of pregnancies with low or no risk of complication can be done by a general practitioner (GP) or midwife [36], but a recent national survey from 2016 showed that in the first 6 months, the majority of women are followed by a gynaecologist in France [37]. The survey further confirmed that insufficient attention is paid to women experiencing psychological disorders during pregnancy. Among women in mainland France who gave birth to a healthy baby, 23.6% reported that they had felt sad, depressed or hopeless for at least 2 consecutive weeks. In 2010, the overall psychological state of French women during pregnancy was described as “quite poor” or “poor” by 8.9% of respondents, yet 93.6% of these women did not have any consultation with a mental health professional; of the remaining women, 1.2% consulted a psychiatrist, and 4.4% consulted a psychologist or psychotherapist. Access to psychiatric or psychological care is difficult in France, and is often associated with high out-of-pocket expenses, even though socio-economically disadvantaged women appear to have the greatest needs in this regard – indeed, the proportion of women suffering from anxiety and/or depression increases significantly with increasing social vulnerability [38]. Against this background, a new law was introduced in France on 7 July 2023, aimed at providing support for couples who experience spontaneous pregnancy loss, by offering reimbursement through the national healthcare system of up to 8 sessions with a psychologist, prescribed by a GP or midwife [39]. So far, this political action has not been followed by a concrete effect in terms of healthcare delivery, since only 5% of practising psychologists are registered with the programme to provide the free care, with the result that out-of-pocket expenses remain high for many women. The law also provides for implementation of a dedicated healthcare pathway, bringing together medical and psychological professionals both in the hospital and the community setting, in a pluridisciplinary approach, to provide enhanced support for women after miscarriage, but roll-out is only due to start in September 2024.

There is thus a compelling need to compile detailed data about the management of psychological disorders in women who have experienced early spontaneous pregnancy loss, especially in France. In this context, the aim of this study is to assess the utility of early, short and accessible psychological care for women who have had an early miscarriage.

## Study objectives

### Study hypothesis

The management in women who have experienced early miscarriage with an early and short psychological care can improve psychological morbidity.

### Primary objective

The primary objective of the study is to establish whether early, short psychological care can improve symptoms of anxiety at 3 months.

### Secondary objectives

The secondary objectives of the study are to establish whether early, short psychological care can improve the following:

1. Symptoms of anxiety at 6 months.
2. Symptoms of depression at 3 months.
3. Symptoms of depression at 6 months.
4. Post-traumatic stress disorder at 3 months.
5. Post-traumatic stress disorder at 6 months.

## Methods

### Study design

This is a prospective, multicenter, randomized, open, controlled, superiority study.

### Participants

To be included, participants must meet all the following inclusion criteria:

1. women who have experienced early miscarriage within the previous 30 days, defined as spontaneous loss of pregnancy before 14 weeks’ gestation;
2. whose pregnancy had been confirmed by blood or urine analysis;
3. regardless of whether or not they had undergone medically-assisted reproduction;
4. age > 18 years;
5. who can speak and read French sufficiently well to participate in the study;
6. affiliated to a social security system;
7. and who provide written informed consent for participation.

The exclusion criteria are as follows:

1. women whose miscarriage is due to ectopic pregnancy;
2. women whose miscarriage is due to molar pregnancy;
3. women with recurrent spontaneous miscarriages (defined according to the WHO criteria as either three or more consecutive miscarriages, or 5 or more miscarriages during the woman’s lifetime (even if she gave birth to a healthy baby between miscarriages));
4. women under any form of judicial protection (e.g. guardianship or curatorship);
5. refusal to provide written informed consent.

### Sample size

The following hypotheses were used to calculate the sample size required for the present study:

- A percentage of women with anxiety at 3 months after randomization equal to 22% in the control group (without early psychological care) [20].
- A percentage of women with anxiety at 3 months after randomization equal to 13% in the intervention group (with early psychological care) (based on the average rate observed in populations with no history of miscarriage).

At an alpha risk of 5% and with power of 90%, in a bilateral situation, the number of subjects needed is 373 per group, i.e. a total of 746 participants. Allowing for 20% of patients lost to follow-up at 3 months after randomization, total accrual of 932 women (466 per group) is planned. Assuming a consent rate of 20%, it is estimated that study information will have to be provided to around 5,000 women in total.

Sample size calculations were performed using NQuery software version 4.0.

### Randomization

Randomization will be performed only after written informed consent has been obtained. Block randomization will be performed using a computer generated randomization algorithm using SAS software (Version 9.4, SAS Institute Inc., Cary, NC, USA). Participants will be assigned to either the intervention group or the control group in a 1:1 ratio, balancing across 2 factors, namely, randomization will be stratified by parity (with vs. without children) and by use of medically assisted reproduction (with vs. without medically assisted reproduction). It is expected that women who have never yet had a live birth and those who become pregnant via medically assisted reproduction may have a more painful psychological experience of miscarriage.

### Recruitment

Recruitment into the study will be performed as follows: Information about the study will be provided through flyers, posters and information leaflets, by the gynaecologists and midwives caring for patients at the time of miscarriage. If the woman accepts to participate, she can contact the clinical research assistant whose contact details are provided on the information leaflet. After verification of the inclusion and exclusion criteria, an inclusion visit will be scheduled with a GP or midwife and will take place by teleconsultation. If, after the inclusion visit, the woman confirms her desire to participate, the informed consent form will be sent to her by post. As soon as the signed informed consent has been returned to the GP or midwife, then the woman is considered to be included in the study, and randomization can take place.

### Study procedures

Randomization will be conducted following return of the signed informed consent form. Following randomization, women will be notified of their allocated group by phone by the GP or the midwife. Within 7 days after randomization, the clinical research assistant will obtain and record study data about the participant and about the miscarriage by phone. During this initial phone call, the STAI Y-A form, as well as the Beck Depression Inventory (BDI) and the Posttraumatic stress disorder Checklist Scale (PCLS) will be completed by the participant.

Two follow-up visits will take place, namely at 3 and 6 months after randomization. These visits will be performed by telephone by the clinical research assistant. For each visit, clinical and demographic data will be recorded (any change in marital status or professional activity, occurrence of any important life events, especially a new pregnancy), and the self-report questionnaires (STAI Y-A form, BDI and PCLS) will be completed again by each participant. Forms may be completed electronically or using paper versions.

### Study interventions

#### Intervention group

Women allocated to the intervention group will have 4 counselling sessions with a psychologist, in addition to the standard of care according to local practice. The first counselling session must be held within 14 days after randomization, and all four counselling sessions must be completed within 2 months after randomization. Each session will last 45 min and will be performed by teleconsultation. The sessions will be based on active listening, expression of emotion surrounding the miscarriage, taking account of each individual’s life circumstances and experiences, and potentially discussing any other important life events that the participant may have found traumatic. Finally, the participants’ resources, possible sources of support and life projects will also be addressed.

#### Control group

Women allocated to the control group will receive standard of care according to local practice.

#### Intervention and control groups

Women in both groups will be encouraged to attend an early consultation with either their GP or a midwife.

### Outcome measures

#### Primary outcome

The State Trait Anxiety Inventory version Y (STAI-Y) consists of two sets of twenty items, which yield scores indicating the level of anxiety the subject has at present (state anxiety, STAI Form Y-A) and the extent to which the person is prone to experience anxiety (trait anxiety, STAI Form Y-B). The Y version was developed to eliminate items that are more specifically bound to depression. In this study, the participants’ feelings of anxiety will be evaluated using the STAI Y-A form (STAI) [40]. The STAI Y-A form only focuses on the psychological, and not the somatic aspects of anxiety. The STAI Form Y-A is a self-reported questionnaire. Each item is scored from 1 to 4 and a sum score of all items is computed. Higher scores indicate greater anxiety. For the purposes of this study, presence of anxiety is defined as a score of 46 or higher.

#### Secondary outcomes

Depression will be evaluated using the Beck Depression Inventory - Second Edition (BDI-II) [41, 42]. The BDI-II is a self-reported index of depressive symptoms experienced in the past 2 weeks. The questionnaire includes 21 items relating to depression symptoms and attitudes. Answers are on a 4-point scale from 0 to 3 (0 = not at all bothered; 3 = severely bothered). The minimum score is 0 and the maximum is 63. Total score indicates that depression is minimal (from 0 to 11 points), mild (from 12 to 19 points), moderate (from 20 to 35 points), or severe (from 36 to 63 points). For the purposes of this study, presence of depression is defined as a score of 19 or higher, i.e. mild, moderate or severe depression.

The possible presence of post-traumatic stress disorder will be evaluated using the Posttraumatic stress disorder Checklist Scale (PCLS) [43]. The PCLS is a short self-report inventory assessing the 3 main syndromes of PTSD, namely intrusion (intrusive feelings and imagery, dissociative-like re-experiencing, flashbacks); avoidance (numbing of responsiveness, avoidance of feelings, situations, and ideas); or hyperarousal (anger, irritability, hypervigilance, difficulty concentrating or sleeping, heightened startling). The PCLS is a questionnaire containing 17 items assessing the intensity of 17 symptoms over the course of the previous month. Responses are ranked from 1 (not at all) to 5 (very often), yielding a total score ranging from 17 to 85. A score of 44 or more identifies PTSD.

#### Clinical data

For all participants, we will record the following data: age, marital status, level of education, occupation, use of psychoactive substances (alcohol, tobacco, cannabis, etc.), personal and family psychiatric history, psychiatric/psychological follow-up (psychiatric hospitalization, psychotropic treatments) and gynecological history (number of pregnancies, number of children, miscarriages (early or not), foetal death, ectopic pregnancy, voluntary interruption of pregnancy, medically assisted reproduction, preterm delivery).

Regarding the miscarriage, the following data will be recorded: whether it was a planned pregnancy, whether the pregnancy was the result of medically assisted reproduction, number of weeks gestation, medical/surgical management for the miscarriage, whether the participant told their entourage about the miscarriage.

### Data analysis

#### Data management

The study will use two distinct databases to separately store research data and contact information. Potentially identifying contact information for the participants, such as name, phone number, and address, will be stored in a separate database unconnected to the research database. In the research database, all participants will be designated by an ID number. Electronic data will be saved on a secure server with restricted access.

#### Statistical analysis

Descriptive analysis will be performed. Quantitative variables will be expressed as the mean ± standard deviation or median (range) and qualitative variables as number (percentage). The primary endpoint (presence of anxiety at 3 months after randomization) will be compared between groups using the chi square or Fisher’s exact test, as appropriate. For the secondary endpoints:

- presence of anxiety at 6 months after randomization, depression at 3 and 6 months, and PTSD at 3 and 6 months will also be compared between groups using the chi square or Fisher’s exact test, as appropriate.
- numerical scores for anxiety at 3 and 6 months, and depression at 3 and 6 months after randomization will be compared between groups using the Student t test.

A p-value of < 0.05 will be considered significant. Statistical analysis will be performed using SAS version 9.4 (SAS Institute Inc., Cary, NC, USA).

### Study progress

The study began enrollment in July 2023 and the target enrollment of 932 women is expected to be completed by July 2025. Data collection is expected to be completed by January 2026.

### Ethical approval

This protocol was approved by the Ethics Committee of Brest Ouest VI (26/01/2023 under the number 22.04193.000135) and by the national data privacy commission (Commission Nationale de l’Informatique et des Libertés) on 29/03/2023. The protocol will be conducted according to the principles of the Declaration of Helsinki and in line with the Guidelines for Good Clinical Practice. The trial is registered with ClinicalTrials.gov under the identifier NCT05653414 (first registered December 15th, 2022).

## Discussion

Psychological care for women who experience spontaneous pregnancy loss in France is considered to be suboptimal, as in many countries. However, the medical community is becoming increasingly aware of this gap in care, which has garnered substantial attention since 2021, and notably since a series of publications on this topic in *The Lancet* [2, 3, 24]. Despite the fact that abundant data in the literature concur that psychological disorders are unfortunately common and often severe after miscarriage, the available literature does not yield a scientifically validated consensus about the optimal management for this specific situation. We therefore believe that there is a compelling need to generate new and scientifically robust results, to provide evidence-based efficacy data regarding the management of psychological disorders in women who have experienced miscarriage, particularly in the French context.

Our working hypothesis is that a short course of early counselling after miscarriage (i.e. within a few weeks immediately following the miscarriage), could help to reduce the proportion of women who suffer from symptoms of anxiety, depression or PTSD after pregnancy loss. The literature suggests that the intensity of these symptoms is at its peak in the first weeks after miscarriage. Therefore, we chose to include in this randomized, controlled trial women who had experienced a miscarriage within the previous 30 days. We also hypothesized that a short course of counselling, with a maximum of 4 sessions of 45 min each, delivered by teleconsultation with a psychologist, would yield a clinically measurable benefit, as assessed by validated instruments (the STAI-Y for anxiety, the BDI for depression and the PCLS for PTSD). According to Barat et al., brief supportive psychotherapy was effective in reducing symptoms of anxiety, depression and grief in women with miscarriage when conducted in the first 24 h of hospitalization, as compared to a control group [31]. Our randomized, controlled trial was designed taking into account the recommendations issued by the WHO regarding flexibility in terms of scheduling of appointments and the care offered, as well as respect for women’s privacy. Accordingly, there will be no out-of-pocket expenses for any of the participants in this study, which will enable the majority of participants to participate, if they so desire, regardless of their socio-economic status or living circumstances.

The inclusion and follow-up visits will be performed by telephone or video-conference, and the intervention itself (psychological counselling) will also be delivered by teleconsultation. Each participant will have a wide range of choices in terms of days and times, in order to adapt to the individual needs and constraints of each (e.g. work schedules, home life etc.), as well as their possible life circumstances (e.g. desire to keep the miscarriage or counselling secret from family members etc.).

Regarding the evaluation of the endpoints, the questionnaires can be completed in paper format and returned by post, or may be completed online or by email. We chose to perform the inclusion visit and to deliver the intervention via telemedicine, in order to limit potential bias related to certain women being more frequently in contact with the healthcare system, or having their own transport, as this could be to the detriment of the inclusion of women in vulnerable socio-economic situations, or who are isolated, or living in rural or underserved areas. We also aim for the study to integrate, as seamlessly as possible, the real-life context of women experiencing miscarriage.

The design and implementation of this study were made possible by the considerable advances in telemedicine delivery made by primary care professionals during the COVID pandemic. In France, teleconsultation is now fully integrated into routine practice, for both patients and healthcare professionals, as a new form of consultation, accessible to the vast majority of people via smartphone, tablet or computer, and allowing for secure exchange of medical information. All the investigators participating in the study are experienced in the delivery of telehealth.

In summary, this study aims to validate the clinical utility of a short course of early psychological care after miscarriage, based on primary care and accessible to all women, with a view to reducing the burden of psychological disorders after early spontaneous pregnancy loss. The results may form the basis for new recommendations for the management of women who experience miscarriage, notably by recommending the involvement of trained psychologists in the management pathway for early pregnancy loss.

## Data Availability

Not applicable.

## List of abbreviations

BDI: Beck Depression Inventory
GP: General practitioner
PCLS: Posttraumatic stress disorder Checklist Scale
STAI-Y: State-Trait Anxiety Inventory version Y
PTSD: Post-traumatic stress disorder
WHO: World Health Organization
